# Spatial Concentrations of Wildlife Attacks on Humans in Chitwan National Park, Nepal

**DOI:** 10.3390/ani10010153

**Published:** 2020-01-16

**Authors:** Aleš Ruda, Jaromír Kolejka, Thakur Silwal

**Affiliations:** 1 Department of Environmentalistics and Natural Resources, Mendel University in Brno, 60200 Brno, Czech Republic; jkolejka@centrum.cz; 2 Department of Parks Recreation and Wildlife Management, Institute of Forestry, Tribhuvan University, Pokhara 44618, Nepal; thakur.silwal@gmail.com

**Keywords:** conservation management, GIS modelling, land use, victims’ density estimation, wildlife attacks

## Abstract

**Simple Summary:**

Wildlife attacks on people in and around protected areas, where the same resources are shared by both people and wildlife, are one of the major conservation challenges in getting effective public support. This empirical study contributed to new understanding of wildlife attacks on people in the landscape of Chitwan National Park (CNP) and its neighborhood based on quantitative survey data from 2003–2013. The results show that the majority of wildlife attacks occurred in forests and cultivated land where people mostly unaware of the behavior of animals were attacked. Mostly the concentration of the attacks occurred in less than one victim per km^2^. A relatively important correlation between land use and animal attacks is visible in the cases of the sloth bear and the leopard. The typical land use structure of sloth bear attacks with more than one victim per 2 km^2^ is given by the dominant percentage of cultivated land, forest, and sandy areas. The remarkably high bear density is due to a relatively high percentage of water bodies, grass land and bush land preferred by the bear. Potential attacks can be reduced through effective management of park and buffer zones, and concerned authorities should also provide education about species-specific behavior of animal attacks.

**Abstract:**

The study was conducted within and adjacent to Chitwan National Park in Nepal (CNP), where several wildlife species are involved in conflicts with humans. We assessed the spatial relationships between the number of victims/km^2^ (=victim density or VD) of attack by wildlife (elephant, rhino, wild boar, sloth bear, leopard or tiger) versus landscape features, including both natural habitat type and land use by humans (e.g., nursery, orchard or cultivated). We identified four levels of VD, ranging from <1 V (victim)/4 km^2^ to >1 V/2 km^2^ for each land use zone, then tested for correlations at one or more of those VD between each pair of wildlife species across different land use types. Our results high correlation for sloth bear and leopard (*r* ≈ 0.8), for all species except elephant and wild boar at VD > 1 V/4 km^2^ (*r* > 0.9) and for leopard vs. rhinoceros (*r* = 0.99) across land use types at 1 V/4 km^2^) indicate some risk-reduction measures. One of them would be division of each buffer zone into three concentric rings, for instance ranging from high-risk adjacent areas to areas of high use by humans, to low-risk where human use is low. This revision would facilitate giving local people more voice in implementing conservation measures and reducing risks.

## 1. Introduction

Since the establishment of protected areas elsewhere in the world, local communities have been restricted somehow or alienated from their traditional practices to use forest resources therein. Additionally, wildlife enters into human settlements and creates havoc by damaging crops, properties, and livestock. In Chitwan National Park (CNP) most poor people living near forests are of indigenous ethnicity and a lower caste, have no or few work opportunities, receive informal education and typically own a smaller piece of land, and survival problems are more acute for them than conservation issues. In places where people and wildlife share the same landscape for resource usage, wildlife leaves negative impacts on people and vice versa [1,2,3,4,5,6,7,8,9,10]. In addition, wildlife damage leads to resentment and people resort to retaliatory killings, even of endangered species. The human population in tiger *(Panthera tigris)* range countries in Asia has doubled in the last few decades and people increasingly come into contact with tigers [5,11] and other dangerous animals, especially in protected areas and their neighborhoods. In Nepal, CNP harbors the largest populations of most endangered and dangerous species, such as the rhino (*Rhinoceros unicornis*), tiger (*Panthera tigris*), sloth bear (*Melursus ursinus*), elephant (*Elephas maximus*), wild boar (*Sus scrofa*), leopard (*Panthera pardus*) and other wildlife, whose populations are on the rise [10]. From the narrow economic human welfare perspective, these endangered species are harmful to the communities and vice versa. It is a generally accepted fact that livelihood activities can undermine the long-term conservation objectives of parks—and this necessarily entails the integration of conservation efforts and activities with socio-cultural and economic objectives and the needs of indigenous people residing around the parks. One previous study [12] suggested that current policy does not address needy communities and that people have exploited buffer zone resources (e.g., arable land, water, timber, and plant biomass). Parts of CNP are inhabited by humans at densities ranging from 1 to 650 inhabitants/km^2^. A buffer zone has been established around each major area of wildlife habitat to protect that habitat and its wildlife from human activities emanating from nearby areas of high human density. Heinen and Mehta [13] stated that the buffer zone in Nepal has granted local participation but the managerial structure remains largely top down with regard to the Park’s Warden regulatory role. Other studies (e.g., tiger: [7,14] and elephant: [8,15]) addressed issues of wildlife attacks on people and recorded injury and death cases in the landscapes of CNP and its neighborhoods. However, there is no simple formula for combining conservation objectives with local needs [16]. According to Parker, Williams, and Turner [17], people’s behavior largely depends on incentives. People have dynamic relationships with wild animals: the outcomes of one or more events may strongly affect people’s attitudes and thereby shape their responses to interactions with a particular species [3,18,19,20]. Some wildlife species, such as large carnivores and mega-herbivores cause considerable harm to people as well as damage to their property [5,9,10,21,22,23]. These damages result in negative attitudes toward the species responsible for the losses [7,9,10,24,25]. An overlap of resource usage occurs between animals and people in the same landscapes. The establishment of protected areas (PAs) has come into direct conflict with traditional linkages and the immediate needs of local livelihoods. Wild animals have been moving out from the parks which has led to an increase in livestock depredation, crop damage, property loss and human injury or death in recent years [26].

Many studies have attempted to address the issue of human-wildlife conflicts (HWCs) to improve the conservation practices in the surrounding landscapes of the park. Conover [18] argued that varieties of management interventions have been adopted to reduce the problems or negative impacts of the wildlife such as killing, and to control the loss of agricultural crops since the beginning of human civilization. However, the existing thinking of human-wildlife conflict management is an activity that seeks to balance the needs of human activity with the needs of wildlife to the mutual enhancement of both. Madden [22] argues that protected areas are increasingly becoming islands of the sea of human-dominated landscapes such as cultivations and developments. In Chitwan National Park, research has mainly focused on the tiger and its prey species [7,9,10,14,27,28,29,30]. The rhino has also been the subject of many studies [31,32]. In recent years, the elephant has also been one of the priority areas of research [8,15]. Besides the focus on these species, there were several studies that addressed socio-economic and policy issues [33,34] and ecosystem and landscapes.

To efficiently manage human-wildlife conflicts including human casualties reduction in CNP, buffer zones were established with active cooperation of local people to protect both wildlife, and human properties, health, and lives. The aim of this measure was also to share revenue generated by conserved wildlife in those buffer zones. So far, natural behavior of wildlife species varies and we expect that the environment of HWCs varies as well. Considering this presumption we focused on identification of characters of environments where HWCs appeared. The aim of the study was to explore relationship between local land use and spatial distribution of wildlife conflicts to improve current management practices. We worked with two research hypotheses:

**Hypothesis** **1** **(H1).** *Wildlife attacks on humans usually take place in a certain type of environment. The types of attack environment, described by the current land use in the area of attacks concentration, may vary depending on the type of attacking animal.*

**Hypothesis** **2** **(H2).** *There is a dependency of attacking animals on similar types of the environment (spatial land use mosaics).*

## 2. Materials and Methods

### 2.1. Study Area

**Figure 1:**
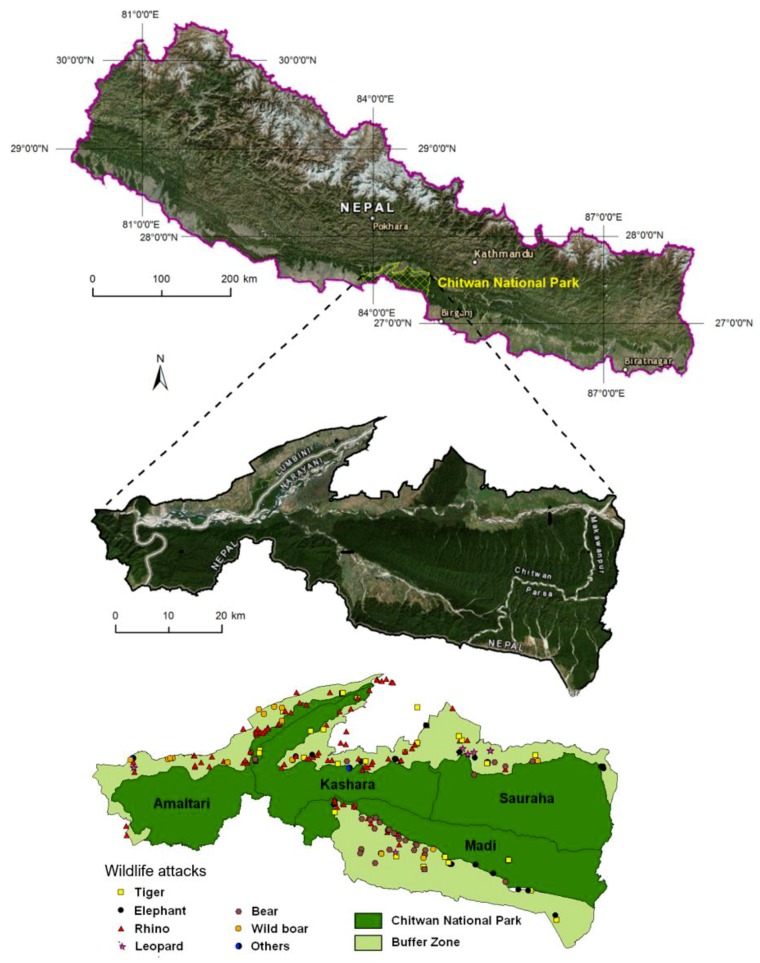
Location of Chitwan National Park on the Nepal–India border.

The study area is situated in CNP, which is located at the foot of the Himalayas in the southern central lowland of Nepal (83°87′79″ E–84°74′30″ E and 27°34′23″ N–27°68′98″ N, Figure 1).

The buffer zone (area: 729.37 km^2^) was proposed to meet the requirements of the local community in order to reduce unwanted pressure on the park. It is a peripheral/surrounding area of the national park intended as a transitional zone between people and wildlife animals established by the government according to National Parks and Wildlife Conservation Act 1973. The buffer zone settlement of CNP consists of 34 Village Development Committees (VDCs) serving as an advisory body to local administration and two Municipalities and it spreads over four districts: Chitwan, Nawalparasi, Parsa and Makawanpur [35]. However, only nine VDCs fall entirely under the buffer zone and the rest of them are only parts of VDCs/municipalities. Altogether 461 human settlements spread across the buffer zone. The location of the buffer zone settlements ranges from less than 1 km to 10 km or more from the park boundary. About 70% of the settlements are located within the range of 1 to 5 km and a few of them are within a distance of 10 km or more from the park boundary. There is a provision to provide 50% of the park’s revenue for local community developments (construction of animal deterrent electric fences, schools, village roads, drinking water, irrigation channels, capacity enhancement training, income generation activities, conservation awareness training, etc.). Forests within the buffer zone have been handed over to local communities as Buffer Zone Community Forests (BZCFs) to meet the requirements of forest products (grass, fodder, timber, leaf litter, fuel-wood) of local communities in order to reduce pressure on the park. The BZCFs are one of the major components of the buffer zone program to develop and fulfil forest resource needs for local communities. Local communities are allowed to use these products for household consumption within the buffer zone but not for sale outside it. CNP was established in 1973 as the first protected area of Nepal and has two distinct physiographic regions: alluvial floodplains and the Terai-Siwalik ecosystem [35]. CNP covered initially an area of approximately 544 km^2^, after enlargement in 1977 its area comprised 932 km^2^ and in 1996 the buffer zone with 750 km^2^ was added. In 2016, the area of CNP and its buffer zone were changed to 952.63 km^2^ and 729.37 km^2^ respectively. A part of the southern boundary of the park follows the Nepal-India border to the Valmiki Tiger Reserve of India. In the north, the park goes through the Brandabhar biological corridor. Chitwan National Park is an internationally recognized UNESCO World Natural Heritage property and it also contains a Ramsar Site—‘Beeshazari and Associated Lakes’ along the Brandabhar biological corridor forest in its buffer zone. In CNP, there are four management sectors: eastern (Sauraha), central (Kashara), western (Amaltari) and southern (Madi). CNP also receives the highest numbers of visitors and generates the highest amounts of revenue mainly from tourism annually; however, it also experiences the highest number of human casualties among all protected areas in the country. That is why this park was selected for study focusing on the assessment of multiple factors causing wildlife attacks, the extent of the injury, people’s perception, and the livelihood condition of the victims.

Vegetation cover of CNP is mostly dominated by forests (85%); however, forests and grassland covers have been converted into shrublands and river bed or sand over the past four decades Grassland coverage in the park decreased from 20 to 12% between 1970 and 2013 [35]. Forests are commonly dominated by sal (*Shorea robusta*) and associated species include Terminalia species, boddhagero (*Lagerstromia parviflora*), tatari (*Dillenia pentagyna*), kyamun (*Syzygium cuminii*), jaamun (*Syzigium operculata*), kadam (*Adina cardifolia*), simal (*Bombax ceiba*) with rhino apple trees (*Trewia nudiflora*), as well as chir pine (*Pinus roxburghii*) in the forested hills of the park [35]. In the floodplain, which includes the tallest grass species in the world (>5 m), characteristic species include kaans (*Saccharum spontaneum*, *S. benghalesis*), reed (*Phragmitis kharka*), giant reed (*Arundo donax*), *Narenga porphyracoma* and shorter species such as *Imperata cylindrica*, *Andropogon spp*., and *Aristida ascensionis*. These grasses are fire and flood resistant and spread rapidly under favourable conditions. grassland and riverine forest support higher ungulate biomass than the sal forest. The floodplain grasslands and riverine forest are attractive habitats for a number of animals.

CNP harbours an exceptionally diverse wildlife population. This park is considered the last surviving example of the natural ecosystems of the Terai region that provides critical habitat for significant populations of several globally threatened species. The park is home to several species of mammals (33%), birds (63%), reptiles and amphibians (34%), fish (65%) and several species of invertebrates of the total numbers recorded in Nepal, which contribute significantly to ecosystem processes in the park [35]. The park harbours not only the world’s largest terrestrial mammal (Asian elephant), but also the world’s smallest terrestrial mammal (Pygmy shrew). Four large herbivores, Asian elephant, rhinoceros, gaur bison, and blue bull also co-exist there [35]. The park and buffer zone habitats support world-endangered species, which are the largest populations of most dangerous animals, such as the rhino (94%), tiger (61%), and elephant (34%) [36,37]. CNP holds the world’s second largest population of Greater One-horned Rhinoceros in the wild. The population increased from about 100 in 1960s to 554 in 2000. As per the rhino count of 2015, the population is 605 in CNP and 645 in Nepal including the translocated population in Bardia National Park and Shukla Phanta National Park from CNP [36]. The Royal Bengal Tiger (*Panthera tigris*) is also found in significant numbers in the Park. The population increased from an estimated 56–60 breeding individuals in 2000 to 120 breeding individuals in 2013. The Sloth Bear population estimated in 1993–1994 was 200–250 and the wild elephant in central Nepal has an estimated population of 40–50 [37].

### 2.2. Data Collection and Processing

During the period of 2003–2013, we collected primary data (data collected by authors and CNP collaborators) on wildlife attacks on humans by rhinoceros, tiger, sloth bear, elephant, wild boar, and leopard. We considered both injuries and fatal cases of wildlife attacks on people in and around CNP. A total of 323 interviews were performed with victims (*n* = 114 only injured victims) and family members of victims or eyewitnesses (*n* = 209). Most family members were eyewitnesses of incidents. The Global Positioning System (GPS) coordinates for each of the 329 incident sites were recorded, including that of the attack site l. GPS points of the victim’s household were not recorded; however, when an attack occurred at home, it was recorded. In addition, the land use category and attack site distance to forest, water body, park boundary, cultivated land and human settlement were also recorded from the incident sites. The topographic map of CNP and buffer zone (with the digital data layers like land use, soil, contour lines, roads, rivers etc.) was obtained from the Survey Department, Ministry of Land Reform and Management, Nepal.

For further processing, GPS points of incident sites were converted into a shapefile format (shp) with the WGS 1984 (Transverse Mercator) coordinate system. In order to determine spatial distribution of victims, Kernel density was calculated using ArcGIS (geographic information system) software. The Kernel function based on the quadratic Kernel function enables a smoothly curved surface fitted over each point to be created [38], which was reclassified according to given parameters (density areas). Kernel density estimation is one of the techniques used to create a surface to indicate the intensity of the events or the phenomenon [39,40]. The Kernel intensity of the event (s) is estimated by the equation:

(1) $$\hat{\lambda_{\tau }}\left(s\right)=\overset{n}{\underset{i}{\sum }}\frac{1}{\tau^{2}}k\left(\frac{\left(s-s_{i}\right)}{\tau }\right)$$

where *s*_1_, …, *s_n_* are locations of the events, *τ* is the bandwidth or the size of the kernel and *k* is the kernel function determining the shape of the kernel.

Based on the spatial distribution of points and the concentration density of attacks generated by KDE, three categories (density areas) of attack intensity were derived. No category with >1 victim per 1 km^2^ occurred. Category 1 represents > 1 victim per 2 km^2^, category 2 represents 1 victim per 2 km^2^ and category 3 represents 1 victim per 4 km^2^. Category number was attached to each (or all) attacking animal(s) where the concentration of its attacks was statistically important for correlation analysis (e.g., Elephant3, All2, etc.).

We calculated Pearson’s correlation coefficient to examine relationship between land use mosaics of HWCs within the category of 1 victim per 4 km^2^ (the only category where all HWCs occurred with the same concentration) (Table 1) and between the dominating land use category (percentage of land usage type) and occurrence of HWCs (density of victims within a land use type) (Table 2). Significance of the correlation coefficient was as follows: *r* = 0.0–0.3 small relationship; *r* = 0.31–0.7 average relationship; *r* = 0.71–0.9 strong relationship; *r* = 0.91–1.0 very strong relationship. The higher correlation values in Table 2 (*r* > 0.3) indicate the environment to which the attacking animals (with respect to the density estimation of the victims) tend. To highlight the higher density of attack concentrations, a category of >1 victim per 4 km^2^ was introduced. It identified areas of higher attack frequency. Grading of other categories of concentrations was proposed based on the calculation of density field estimation and suitably comparable incidences of attacks between individual animals. Using topological overlay (superimposing of layers) of different density areas and land use categories a percentage (1% or more) of different land use categories within density areas of attacking animals was calculated and graphically visualized (diagrams and maps).

## 3. Results

### 3.1. Concentration of HWCs

**Figure 2:**
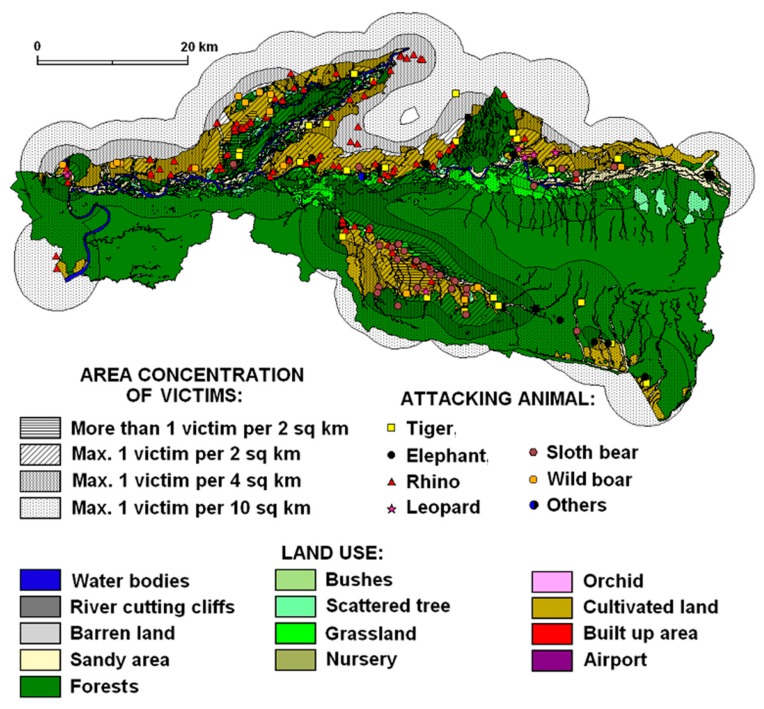
Spatial distribution of wildlife attacks on humans in Chitwan National Park.

A total of 329 attacks that ranged from 16 to 48 people per year (on average 30) were recorded during the decade (2003–2013). Attacks fluctuated annually but show an increasing trend in recent years. The highest (*n* = 48) and lowest (*n* = 16) number of attacks occurred in 2010 and 2006. There was considerable variation between the years, with a mean of 30 attacks (st. dev. = 3.13) every year. The number of attacks fluctuated but there was an increasing trend in attacks since 2008. Compared to 2003, the number of attacks almost doubled in 2010 and 2012. Generally, 89% of attacks were located outside the park boundary, 74% of attacks were recorded in the 1 km buffer zone within the park boundary and 37% of the attacks happened in the BZCFs. Attacks were concentrated mostly on the edge of a forest or on cultivated land near buffer zone forests (Figure 2). The results show that the majority of the wildlife attacks occurred in forests and cultivated land where people mostly unaware of animals’ behaviour were attacked.

We identified four levels of victim density (V = victim), ranging from <1 V/4 km^2^ to >1 V/2 km^2^ for each land use zone (Figure 2) but category 1 V/10 km^2^ has a marginal sense because it includes area of all recorded attacks. Then we tested for correlations at one or more of those victim densities between each pair of wildlife species across different land use types: (a) for sloth bear and leopard *r* ~ 0.8 across all victim densities and land use types; (b) concerning correlations between victim densities for each pair of species across all land use types, all species except elephant and swine had *r* > 0.9 with the other species at victim densities >1 V/4 km^2^; (c) for leopard vs. rhino across land use types: *r* = 0.99 for attack levels of 1 V/4 km^2^. More than 1 victim per 2 km^2^ was identified on mostly inhabited cultivated land (49% of all incidents) followed by 31% on forest land, 6% on sandy areas, 5% on grassland, 4% on bush/shrub land and just a few on orchard and barren land. The high correlation values in Table 1 illustrate high similarity of the environments (land use mosaics) of different HWCs except wild boar and elephant (bold entry), where it is evident that they are attacking in other types of environments. Our results (Table 2) confirmed a relatively important correlation (Pearson´s correlation coefficient) between land use and animal attacks, which is visible in the cases of sloth bear (where bear1 is the highest territorial concentration of victims caused by bears) and leopard (where leopard2 is the relatively highest concentration of victims caused by leopards). Both these animals have their operation range (finished with attacks) in open cultivated countryside (*r* is around 0.8). Probable explanation can be related to generally small size of these animals and their necessity to have a wider territorial range when moving in an area. This also coincides with the most densely populated type of land use, which is the cultivated land supplemented by scrubs.

### 3.2. Attacking Animals

#### 3.2.1. Rhinoceros Attacks

**Figure 3:**
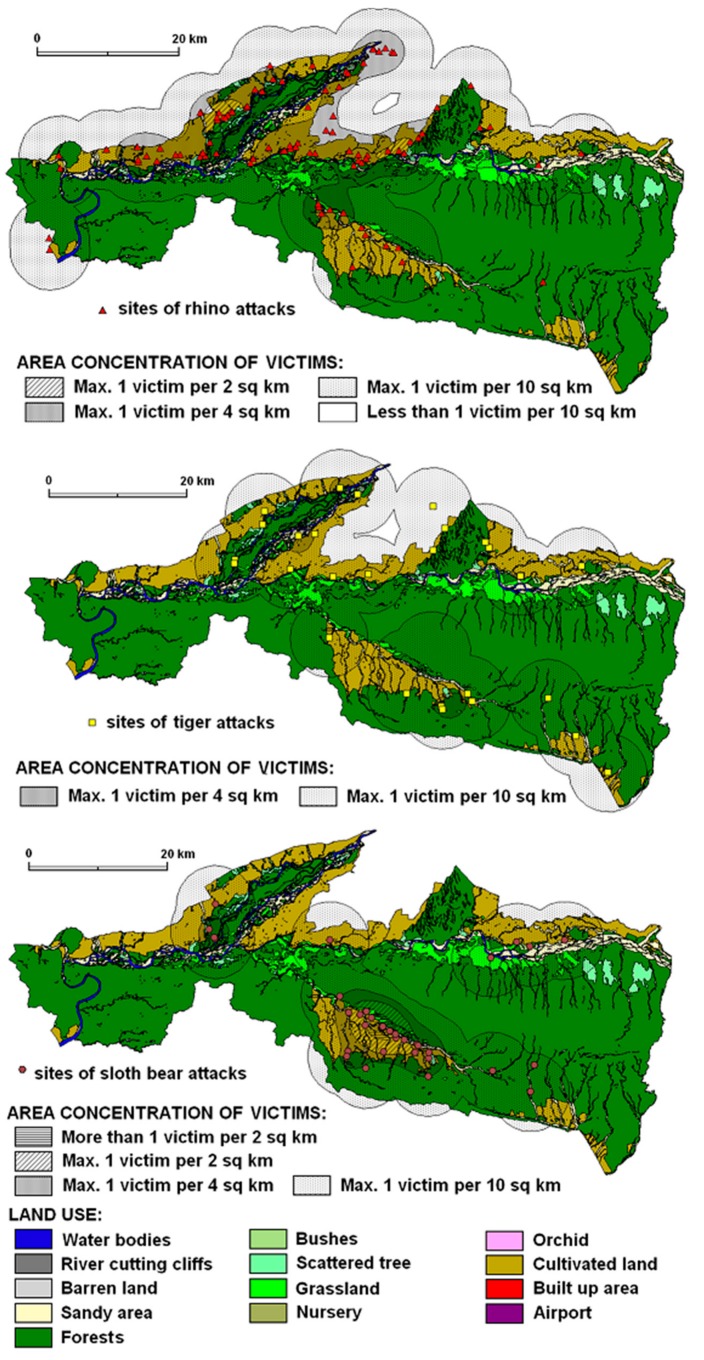
Density areas of recorded attacking animals (rhinoceros, tiger and sloth bear) within different land use categories.

**Figure 4:**
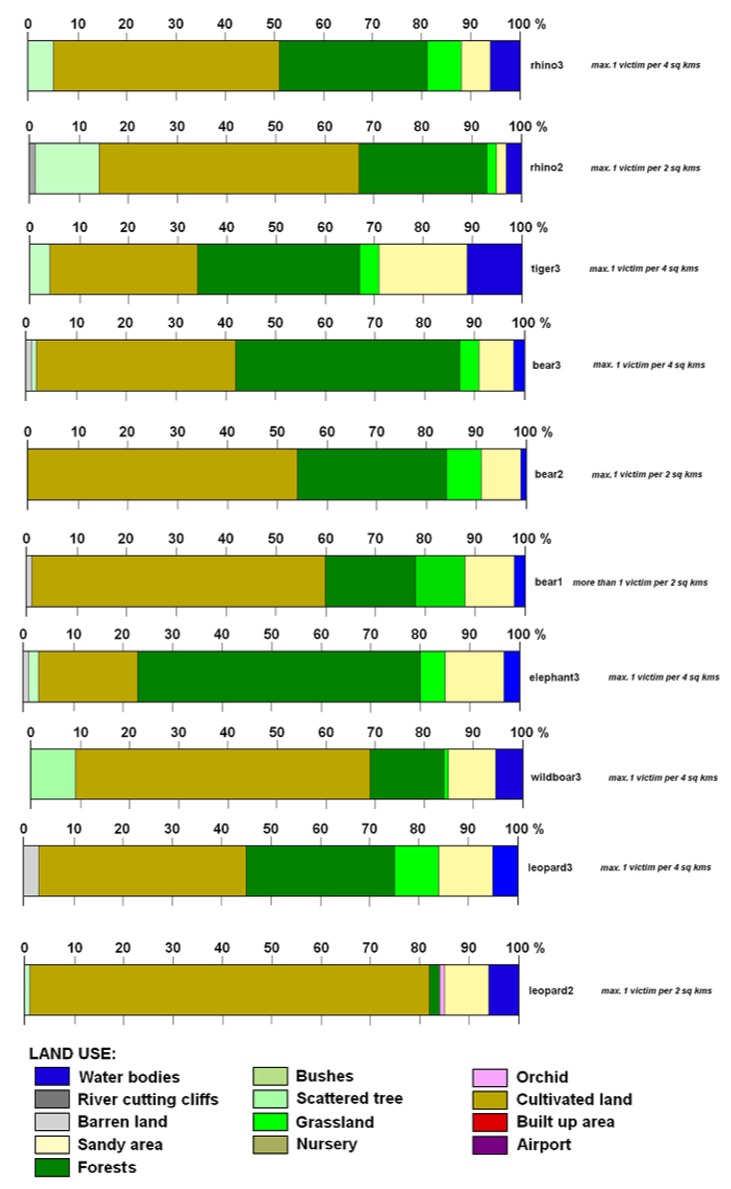
Share (1% or more) of different land use categories within operational areas of attacking animals.

The locations of rhinoceros attacks reflect their wider range in the peripheral landscapes of Chitwan National Park. The occurrence of rhino attacks was the highest in numbers (*n* = 126) or approximately 40% of recorded victims between 2003 and 2013. The attack concentrations reached a maximum of 1 victim per 4 km^2^ on cultivated land, forest land, shrub land and water bodies across the territory (Figure 3). However, the exceptional cases of max. 1 victim per 2 km^2^ occurred in the Kashara and Amaltari sectors. The land use structure of rhinoceros attacks is given by the highest percentage of cultivated land, e.g., rhino2 (54%) and rhino3 (45%). However, the percentage of rhino3 and rhino2 was 29% and 25% respectively in the forest area followed by bush/shrub land and sandy areas (Figure 4). The rhinoceros attacks occurred in almost all land use categories in and around CNP. However, a remarkable feature is the relatively high percentage of barren land and water bodies and important percentages of river cliffs and orchards.

#### 3.2.2. Tiger Attacks

The territorial concentration of attacks featured a maximum of 1 victim per 4 km^2^ (relatively sparse concentration) in forested areas, cultivated land, waterholes, bushes and grassland. The typical land use structure of tiger attacks is given by the balanced percentage of forest (34%) and cultivated land (30%), and sandy areas (17%) (Figure 3 and Figure 4). Basically, tigers prefer open grassland, riverine forest, and look for three things (cover, water and prey) in abundance when looking for a habitat.

#### 3.2.3. Sloth Bear Attacks

The typical land use structure in the areas of sloth bear attacks is given by the dominating percentage of cultivated land (58%), forest (17%), grassland and sandy areas (each 10%). Land use differences in areas with different concentrations of victims are negligible. Subtle differences can be seen when comparing areas with the highest concentration of victims (more than 1 victim per 2 km^2^ recorded only within this animal category) with other action ranges of the sloth bear. The highest concentrations of attacks ranging from 1 victim per 4 km^2^ to >1 victim per 2 km^2^ (Figure 3 and Figure 4) were recorded in forests or close to a forest (cultivated land).

#### 3.2.4. Elephant Attacks

**Figure 5:**
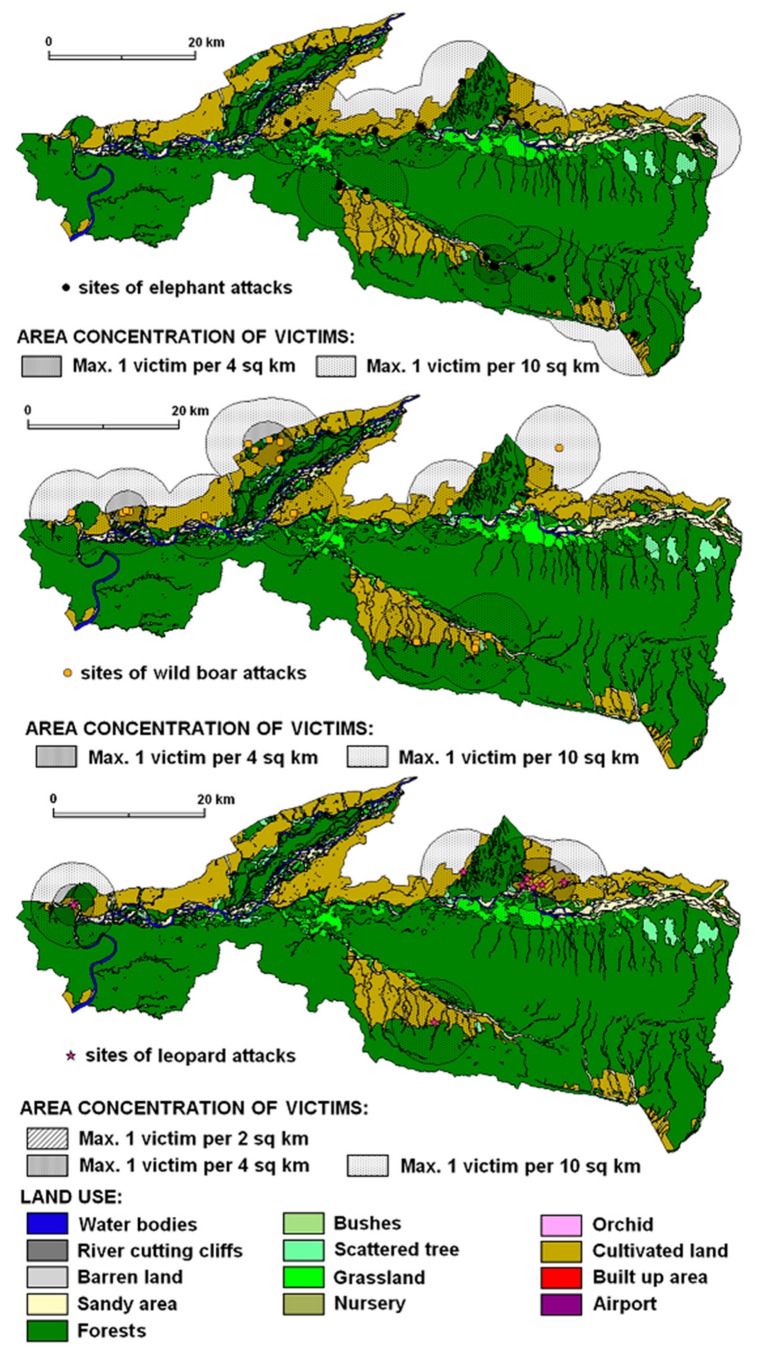
Density areas of recorded attacking animals (elephant, wild boar, and leopard) within different land use categories.

Elephant attacks concentration evidenced a maximum of 1 victim per 4 km^2^ in the landscape (Figure 5). Based on ANOVA results (*p* = 0.018), the land use structure of elephant attacks differs from the land use structure of other attacking animals. Especially the large percentage (58%) of HWCs in forest indicates woodland as the dominant attacking environment which is similar to sloth bear attacks within the same area of attacks (Figure 4).

#### 3.2.5. Wild Boar Attacks

The concentration of wild boar attacks (Figure 4) showed a maximum of 1 victim per 4 km^2^ in the landscape of Chitwan National Park and its neighbourhoods mostly in cultivated land (60%). Attack locations and the percentage of cultivated land together present attack sites as close-to-forest in open areas, i.e., in forest-open land and cultivated land in the buffer zone where human activities and wild boar movements occur (Figure 5).

#### 3.2.6. Leopard Attacks

Leopard attacks represent a small percentage of victims with minor injuries in the landscapes of Chitwan National Park. However, the concentration of attacks showed a maximum of 1 victim per 2 km^2^ in the Sauraha sector (Figure 5). Leopard attacks operational zone was identified mainly in cultivated land (82%) which is followed by sandy areas (10%) and grassland (5%) (Figure 4). 

## 4. Discussion

The widening of the operational area (from high to low victim density) shows the growing percentage of the forest as a scene of attacks. That means the forest is the key area where animal-human conflicts occur and people-victims move in cultivated land not usually close to neighbouring forests. Our research also shows that in the open-air environment (about 2/3 of the land where attacks occurred), with the predominance of cultivated land simulating natural wet areas (e.g., irrigated rice pads) and grassland (unitary mature agricultural crops before harvest), the majority of attacks take place. Basically, rhinoceroses prefer marshy land or water bodies to wallow and open grass land habitat [34]. They prefer to forage in grasslands and eco-tones (grasslands interspersed with mixed forests) and riverine (alluvial) mixed forests for resting [35]. Tigers inhabit diverse environments of the forest, open grasslands near water bodies, which corroborates the previous results [7,29]. Tigers in lowland forests prefer to inhabit the slopes of the Siwalik range and in rich grasslands and riverine forests they inhabit a series of valleys. Sloth bears need a forest in their background and come down to the lowland during the dry season and back to the upland in the wet season [37], which confirms our findings. In some areas of CNP and its neighbourhoods, sloth bears are more feared than tigers, due to their unpredictable nature. They are omnivores and come to forage in cultivated land. The relatively large percentage of forest (58%), cultivated land (19%), sandy area (11%) and grassland (4%) in elephant attacks operational zone shows the fact that elephants do not leave the woodland for large distances. Attack locations and percentage of open land together present attack sites as close-to-forest in open areas and close-to-open areas in forest locations, i.e., in the forest-open land buffer zone where human activities and elephant movements occur. Pant et al. [8] and Koirala et al. [15] state that elephants forage in forested areas and feed on cultivated crops such as bananas, rice, wheat, maize and sugarcane, which causes increasing conflicts with humans across their territory. We also indicated that wild boar attack operational zone is mostly found in cultivated land (60%) and based on GON [35] it shows the fact that wild boars live in the forest for their shelter, but commonly come to feed in cultivated land. On the contrary, the balance percentages of the sandy area, bushes (each 9%) and water bodies (5%), representing open or semi-open areas, showed such a relatively small animal´s preference for areas with long visibility. In relation to topological overview, a very large percentage of cultivated land (82%) in leopard attacks operational zone shows the fact that leopard prefers the fringe area in the forest (29%) for getting shelter close to built-up areas. 

## 5. Conclusions and Management Implications

**Figure 6:**
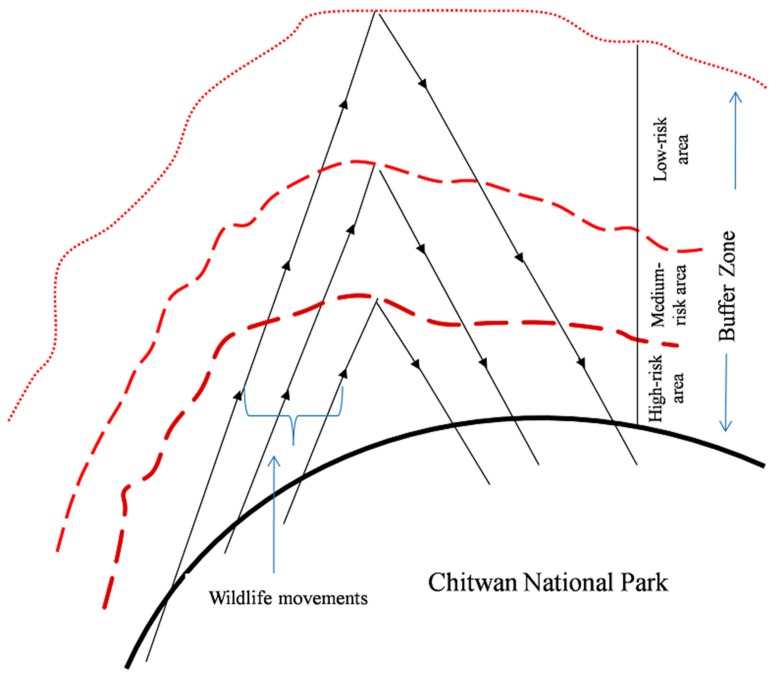
Sketch map of wildlife movement in human-dominated landscapes of Chitwan National Park (the arrows show wildlife movement patterns to and from the park).

Existing practices of buffer zone delineation need to be redesigned with an emphasis on the possible risks of wildlife damages. Alternative ways of conceptualizing the interactions between wildlife and local communities offered new perspectives of the buffer zone delineation. To propose the category of considering wildlife pressure on human-dominated landscapes a new solution was sketched out (Figure 6).

The risk-reduction measures within the buffer zone around each area of protected habitat have traditionally been managed in the same way throughout each buffer. Our results indicate that risk-reduction measures would be more effective and efficient if each buffer zone was divided into three concentric rings, for instance ranging from high-risk adjacent to areas of high use by humans, to low-risk where human use is low. Each part should be managed by different level of surveillance and security measures (e.g., electronic barriers, photo traps, acoustic warning system, mobile phone alert system, etc.). The easiest way is to build watch towers in the most risky areas with trained staff. This revision would facilitate giving local peoples more rights in planning and implementing conservation measures and in reducing risks (e.g., improving organization of self-protection). Obviously, there is an inverse relationship between wildlife pressure (movements outside the park) and distance from the park boundary. Most of the incidents occurred close to the park’s “high-risk areas” within 1 km. If the buffer zone was categorized based on wildlife movement, wild animals would enter the human-dominated landscapes anywhere where current electric fence does not work properly to search for suitable foods and breeding places. Those animals make a short journey and return to the park, which accounts for twice the movements in the same areas. The wild animals frequently use the closest areas for both times, up and down, even occasionally reaching places far from the park boundary. The sketch coincides with a previous study conducted by Budhathoki [33] reporting that people perceived the inverse relationship between the proportion of receiving benefits from the buffer zone program and the distance of settlements located from the park boundary. This might be due to the lack of representative people from the more affected areas in the decision-making level of the Buffer Zone Management Committee. Budhathoki [33] also argued that many beneficiaries of the buffer zone programs were the rich, the elite and BZUC office bearers due to their access to the decision-making level. Most of the inhabitants belong to indigenous ethnic groups and poor households close to the forests have less formal education, thus get fewer opportunities [1]. So, the nearest areas have to bear more than 50% of the total wildlife pressure and can be classified as high-risk areas, followed by medium-risk areas, and then low-risk areas. The proposed participatory sketch map might be an example for further delineation of buffer zones based on HWCs cases for more effective program planning and resource allocation (better education, higher awareness and better security self-organization). The policy makers and park authorities should distinguish between human–wildlife impacts and human–human conflicts of personal interest and be explicit about the different interests involved in conflicts, which could offer new approaches for easing human-wildlife coexistence.

The wildlife damages, including human casualties, can never be controlled but they should be brought to a limit that people are ready to accept [41]. Reducing casualties should be the first management priority over killing animals, building fences, or transporting them to the zoo.

## Figures and Tables

**Table 1:** Pearson’s coefficient of correlation between percentage of specific land use mosaics of human-wildlife conflicts (HWCs) with a maximum of 1 victim per 4 km^2.^

Animals	Sloth Bear	Elephant	Leopard	Rhinoceros	Tiger	Wild Boar
Sloth Bear	x	0.9019	0.9547	0.9456	0.9370	0.7801
Elephant		x	0.7636	0.7282	0.8739	0.4575
Leopard			x	0.9927	0.9356	0.9047
Rhino				x	0.9058	0.9287
Tiger					x	0.7699
Wildboar						x

**Table 2:** Pearson’s correlation coefficient (*r* > 0.3 shown in bold) between areas in sq. km of different concentrations of HWCs and land use category (**animal name_1**: more than 1 victim per 2 km^2^, **animal name_2**: max. 1 victim per 2 km^2^, **animal name_3**: max. 1 victim per 4 km^2^).

Land Use Category	Density of Victims by Chosen Groups of Attackers												
	All3	All2	All1	Bear3	Bear2	Bear1	Elephant3	Leopard3	Leopard2	Rhino3	Rhino2	Tiger3	Wildboar3
Sandy area	0.0903	0.0720	0.0654	0.0694	0.0771	0.1015	0.1194	0.1050	0.0921	0.0623	0.0152	0.1771	0.0959
Forest land	0.4049	0.3150	0.3154	0.4529	0.3010	0.1779	0.5771	0.2986	0.0168	0.2948	0.2568	0.3336	0.1543
Bush/shrub land	0.0289	0.0377	0.0419	0.0138	0.0002	0.0010	0.0252	0.0258	0.0070	0.0478	0.1253	0.0443	0.0897
Cultivated land	0.3483	0.4581	0.4917	0.4016	0.5365	0.7911	0.1973	0.4241	0.8170	0.4571	0.5410	0.2996	0.5948
Grass land	0.0738	0.0662	0.0528	0.0413	0.0677	0.1009	0.0457	0.0873	0.0045	0.0722	0.0177	0.0413	0.0056
Barren land	0.0027	0.0053	0.0027	0.0063	0.0052	0.0042	0.0069	0.0032	0.0000	0.0032	0.0137	0.0000	0.0001
Built up area	0.0006	0.0007	0.0000	0.0000	0.0000	0.0000	0.0000	0.0007	0.0009	0.0011	0.0000	0.0011	0.0000
Nursery	0.0000	0.0000	0.0000	0.0000	0.0000	0.0000	0.0000	0.0000	0.0000	0.0000	0.0000	0.0001	0.0000
Orchard	0.0005	0.0008	0.0056	0.0005	0.0011	0.0008	0.0009	0.0010	0.0051	0.0006	0.0005	0.0001	0.0003
Water bodies	0.0489	0.0420	0.0295	0.0142	0.0113	0.0225	0.0276	0.0542	0.0566	0.0601	0.0298	0.1068	0.0591
River cutting/cliffs	0.0002	0.0002	0.0000	0.0000	0.0000	0.0000	0.0000	0.0000	0.0000	0.0005	0.0000	0.0000	0.0001
Scattered trees	0.0009	0.0017	0.0000	0.0000	0.0000	0.0000	0.0000	0.0000	0.0000	0.0000	0.0000	0.0000	0.0000
Airports	0.0001	0.0003	0.0000	0.0000	0.0000	0.0000	0.0000	0.0000	0.0000	0.0003	0.0000	0.0000	0.0000
